# A Flexible and Polarization-Insensitive Metasurface Harvester Featuring a Dual-Ring Unit with a T-Shaped-Gap Outer Ring for Microwave Power Transfer

**DOI:** 10.3390/mi17030319

**Published:** 2026-03-04

**Authors:** Zhonglin Li, Tianxin Ma, Qian Yu, Yu Zhao, Zhuozheng Wang, Xu Liu, Tao Chen

**Affiliations:** 1 School of Physics and Optoelectronic Engineering, Beijing University of Technology, Beijing 100124, China; lizhonglin@emails.bjut.edu.cn (Z.L.);; 2 School of Information Science and Technology, Beijing University of Technology, Beijing 100124, China

**Keywords:** wireless power transfer, flexible metasurface, polarization-insensitive, microwave energy harvesters

## Abstract

This paper proposes a flexible and polarization-insensitive metasurface (MS) operating at the 5.8 GHz band for electromagnetic energy harvesting. The proposed MS unit features a top-layer dual-ring resonator with a T-shaped gap and a bottom cross-shaped coplanar waveguide (CPW), fabricated on a flexible polyimide substrate. To elucidate the physical mechanism of energy capture, an equivalent circuit model is established based on transmission line theory. Expressions for the total input impedance are derived, revealing the quantitative relationship between the structural parameters and the impedance-matching condition. The simulation results validate this theoretical model and show that the structure achieves an absorption efficiency of 97.5% and a harvesting efficiency (HE) of 86.6% at 5.72 GHz. The conversion efficiency remains above 50% over a wide range of incident angles, and the HE exhibits minimal variation within a polarization angle range of 0–90°. Experimental results indicate that the MS reaches a maximum HE of 73.2%, maintains over 40% efficiency under large-angle incidence, and achieves more than 65% HE across various curved surfaces. With its mechanical flexibility, polarization insensitivity, and simplified manufacturing, this MS harvester provides a reliable and scalable power solution for wireless power transfer applications.

## 1. Introduction

Wireless power transfer (WPT) technology, which eliminates the limitation of using cables to power devices [1], has recently emerged as a pivotal technology. By providing contactless electrical power transmission, WPT offers convenient, safe, and versatile power-supply solutions for industrial and commercial applications, including Internet-of-Things devices [2], sensors [3], biomedical implants [4], and wearable electronics [5].

Leveraging the exceptional electromagnetic-wave-manipulation capabilities of MS [6,7,8], MS-based energy harvesters have garnered significant interest in the field of wireless power conversion in recent years [9,10,11,12,13]. Furthermore, related research has extended into their advanced manufacturing processes and practical application environments [14,15]. However, their rigid construction hinders their application in complex scenarios, such as those with non-planar receiving surfaces [16,17,18].

MS based on flexible materials can exhibit excellent HE under various conditions, significantly enhancing their applicability in challenging environments. Substrates such as conductive fabrics [19], PDMS [20], and neoprene [21] have been employed to achieve flexibility. Unfortunately, their high loss tangent results in substantial dielectric loss. An alternative approach involves embedding metallic vias within the unit cells to enhance power collection [22,23,24]. Furthermore, a significant challenge remains in balancing mechanical compliance with electromagnetic performance; existing flexible harvesters often sacrifice polarization stability or operating bandwidth to accommodate structural deformation [25], limiting their reliability in dynamic practical scenarios.

To overcome these limitations, this paper proposes a flexible MS harvester operating at 5.8 GHz. The device captures energy through a top radiation patch and transmits it to the load on the back CPW structure through electromagnetic coupling. The MS is fabricated on a flexible polyimide substrate with a copper radiator. Its performance, particularly in terms of its performance under bending, was analyzed using the CST Studio Suite (CST) frequency domain solver. The results indicate that the proposed structure demonstrates high HE within the target frequency band, while also exhibiting polarization insensitivity and a wide angle of incidence. A prototype of the harvester was fabricated and tested, and the measured results agree well with the simulations.

## 2. Design and Principle

**Figure 1:**
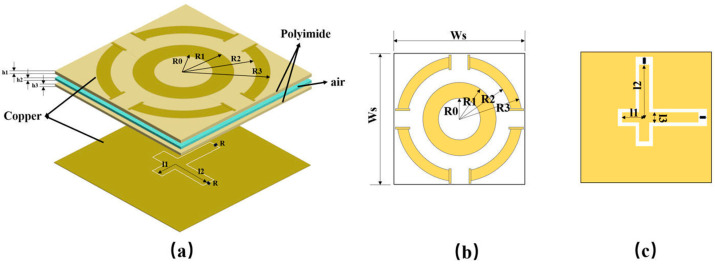
Metasurface (**a**) topological structure, (**b**) upper surface, and (**c**) lower surface.

This section details the design of an MS unit cell with a concentric split-ring geometry, as depicted in Figure 1. The unit cell features a sandwich-type dielectric configuration, consisting of two polyimide (PI) layers separated by a central air gap. Both PI layers have a permittivity ε_r_ of 3.5 and a loss tangent tan δ of 0.003. The air gap has a thickness of 0.5 mm.

The top metal pattern consists of an inner annulus, defined by inner radius R_0_ and outer radius R_1_, and an outer split ring, defined by inner radius R_2_ and outer radius R_3_. The bottom layer is a solid grounding plane with two orthogonal feed lines. Each feed line has a short arm of length l_1_ and a long arm of length l_2_, with a lumped load resistor (R) connected at the end of the long arm. The top metallic pattern captures incident electromagnetic energy and couples it to orthogonal feedlines on the bottom layer, which then deliver the energy to the load resistor.

The side length of the square unit cell is Ws. All metallic parts are made of copper with a thickness (h_0_) of 50 μm and an electrical conductivity (σ) of 5.8 × 10^7^ S/m. The dimensions are chosen as follows: Ws = 25 mm, R_0_ = 4 mm, R_1_ = 7 mm, R_2_ = 10 mm, R_3_ = 12 mm, h_1_ = 1 mm, h_2_ = 0.5 mm, h_3_ = 1 mm, l_1_ = 7 mm, l_2_ = 11 mm, and l_3_ = 2 mm.

**Figure 2:**
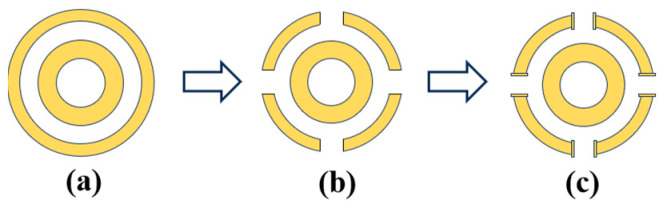
Designing procedure of the MS units. (**a**) Double concentric ring structure, (**b**) Step 1, and (**c**) Step 2.

**Figure 3:**
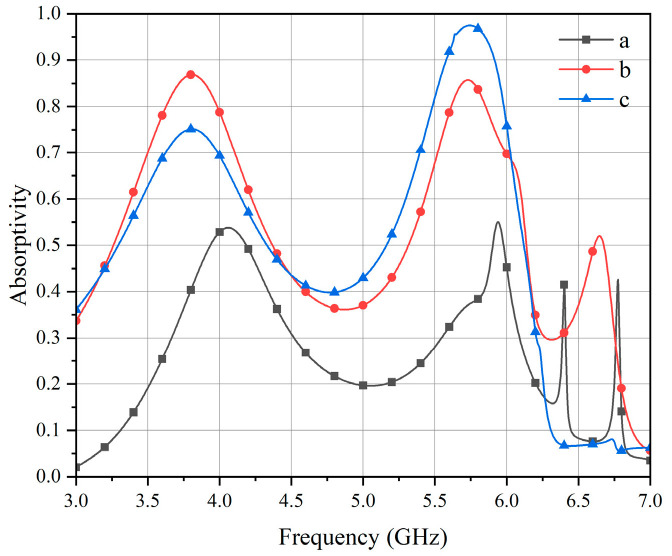
Absorption rates of different unit structures.

The design procedure of the unit cell is illustrated in Figure 2, beginning with a centrosymmetric double concentric ring structure with low mutual coupling, as shown in Figure 2a. To quantify the performance gain of the proposed topology, the loaded Q-factor and half-power bandwidth were evaluated for each state. The initial continuous ring exhibits a high loaded Q-factor of 9.58 and a narrow full-width at half-maximum (FWHM) of 0.62 GHz, leading to a severe impedance mismatch and a limited peak absorption of 54% at 5.8 GHz. Subsequently, narrow gaps were etched into the outer ring to further diminish coupling between elements, as depicted in Figure 2b. This gap structure reduces the Q-factor to 5.95, thereby improving the absorption to 85.7% and broadening the FWHM to 0.964 GHz. Finally, square patches were added at these gaps to enhance the local resonance within the unit, yielding the T-shaped topology presented in Figure 2c. This T-shaped gap structure accurately fine-tunes the loaded Q-factor to 5.81, achieving perfect critical coupling with the free-space wave impedance. As demonstrated by the corresponding absorption rate in Figure 3, this precise impedance-matching eliminates residual reflection, boosting the peak absorption to 97.5% and further expanding the operational FWHM bandwidth to 0.988 GHz. This final unit cell preserves centrosymmetry, thereby ensuring polarization-insensitive performance, which led to its selection for the MS.

**Figure 4:**
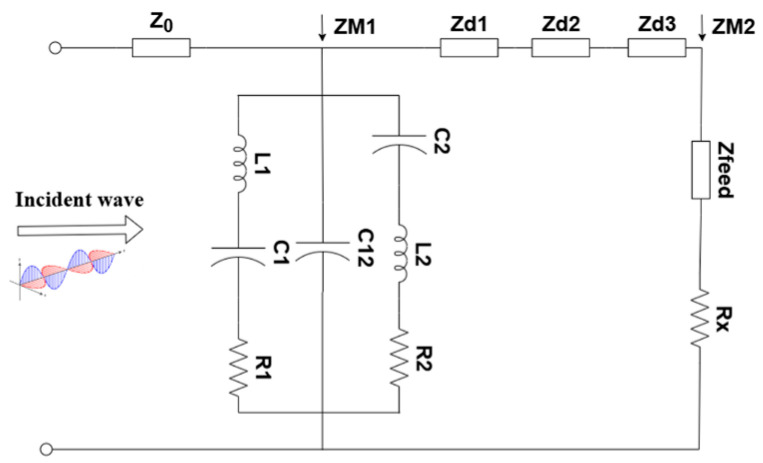
Equivalent circuit of energy-harvesting MS.

**Figure 5:**
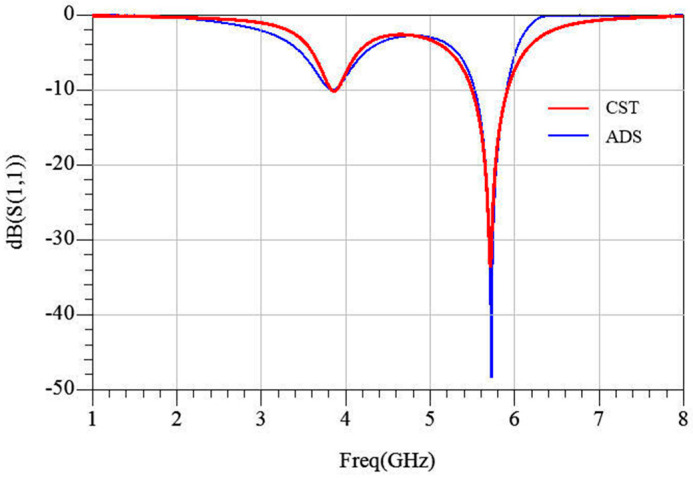
Reflection coefficient of full wave simulation and circuit simulation.

Based on equivalent-circuit theory [26], the MS is modeled by the lumped network shown in Figure 4. The free-space wave impedance Z_0_ (377 Ω) is connected in series with the metal-layer impedance, Z_M1_. For the top radiation patch Z_M1_, C_1_ and C_2_ represent the coupling capacitance between the outer-ring patches and between adjacent units, respectively; L_1_ and L_2_ respectively represent the magnetic energy stored in the inner and outer rings; and C_12_ represents the gap capacitance between the rings. Metallic loss is modeled by resistances R_1_ and R_2_, accounting for the finite conductivity of the copper patch. The dielectric–air–dielectric sandwich-type configuration is represented by three cascaded transmission-line sections with characteristic impedances Z_d1_, Z_d2_, and Z_d3_, while the CPW is described by its characteristic impedance, Z_feed_. The load is represented by a resistance, Rx. The resulting network was optimized in a Keysight Advanced Design System (ADS). The circuit-simulated reflection coefficient is plotted in Figure 5, showing excellent agreement with the full-wave results, thereby validating the equivalent model. This circuit can be used to qualitatively analyze the impedance-matching between the unit cell and free space under varying geometrical parameters.

Based on the equivalent circuit model illustrated in Figure 4, the input impedance of the MS can be derived. The equivalent impedance Z_3_, which represents the input impedance looking into the bottom dielectric layer loaded by the bottom metal structure Z_M2_, is expressed as follows:

(1) $$Z_{3}=Z_{d3}\frac{Z_{M2}+jZ_{d3}\tan(\beta_{3}d_{3})}{Z_{d3}+jZ_{M2}\tan(\beta_{3}d_{3})}$$

Considering the free-space characteristic impedance Z_0_, the equivalent impedance Z_2_ is obtained by transforming Z_3_ through the air gap transmission line. This is given by the following:

(2) $$Z_{2}=Z_{0}\frac{Z_{3}+jZ_{0}\tan(\beta_{2}d_{2})}{Z_{0}+jZ_{3}\tan(\beta_{2}d_{2})}$$

Similarly, the equivalent impedance Z_1_ is calculated by transforming Z_2_ through the upper dielectric layer, which can be written as follows:

(3) $$Z_{1}=Z_{d1}\frac{Z_{2}+jZ_{d1}\tan(\beta_{1}d_{1})}{Z_{d1}+jZ_{2}\tan(\beta_{1}d_{1})}$$

Finally, taking the top radiation patch impedance Z_M1_ into account, the total input impedance Z_in_ of the MS equivalent circuit is defined as the parallel combination of Z_M1_ and Z_1_:

(4) $$Z_{in}=\frac{Z_{1}Z_{M1}}{Z_{1}+Z_{M1}}$$

where Z_di_ represents the characteristic impedance of the medium, and β_i_ denotes the phase constant, expressed as follows:

(5) $$Z_{di}=\frac{Z_{0}}{\sqrt{\varepsilon_{ri}}}$$

(6) $$\beta_{i}=\frac{2\pi f\sqrt{\varepsilon_{ri}}}{c}$$

Additionally, Z_M1_ and Z_M2_ represent the impedances of the top and bottom metal structures, respectively, and are given by the following:

(7) $$Z_{M1}=\frac{1}{\frac{1}{\left(R_{1}+\frac{1}{j\omega C_{1}}+j\omega L_{1}\right)}+j\omega C_{12}+\frac{1}{\left(R_{2}+\frac{1}{j\omega C_{2}}+j\omega L_{2}\right)}}$$

(8) $$Z_{M2}=Z_{feed}+R_{x}$$

To achieve high absorptivity, the input impedance of the MS must match the free-space wave impedance. Simultaneously, to enhance energy HE, proper impedance-matching between the load resistance and the MS is also essential.

**Figure 6:**
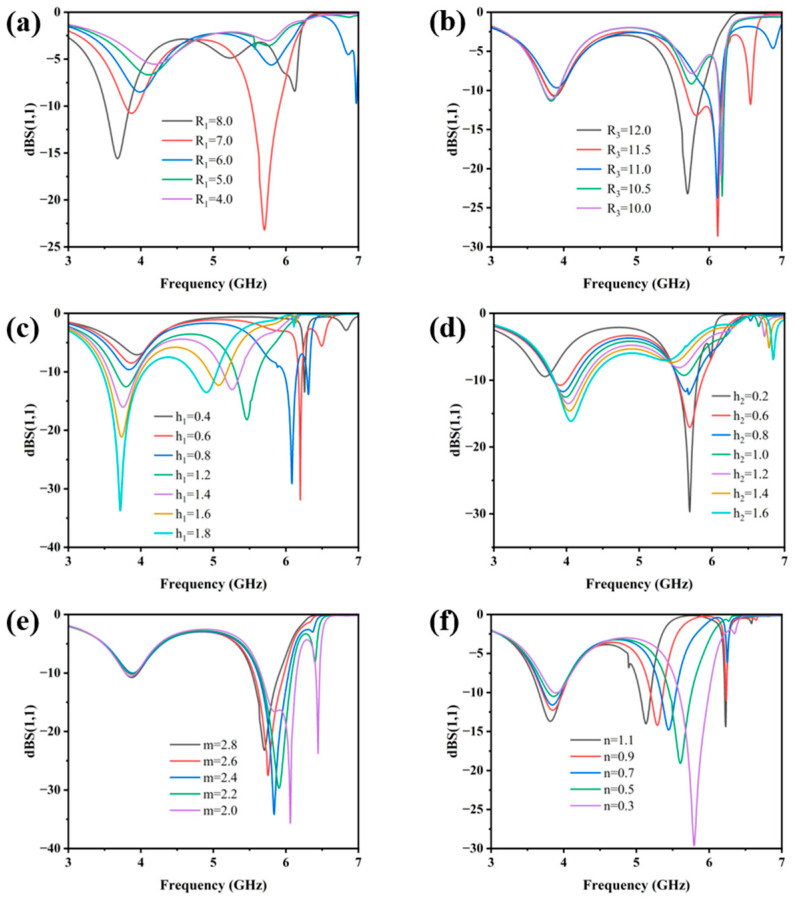
S11 variation diagram of the MS energy harvester when (**a**) R1, (**b**) R3, (**c**) h1, (**d**) h2, (**e**) m and (**f**) n changes.

**Figure 7:**
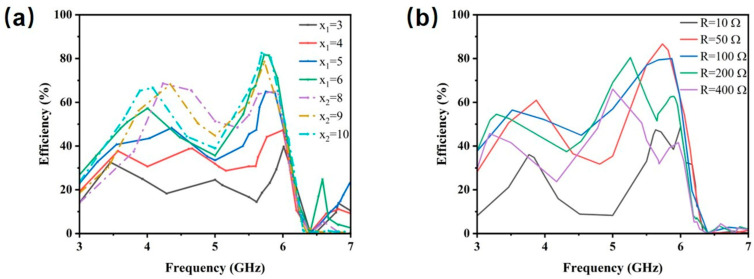
Harvesting efficiency variation diagram of the MS energy harvester when (**a**) x x_1_ and x_2_ (**b**) R changes.

Figure 6 and Figure 7 display the simulated reflection coefficient (S11) and HE of the MS for different structural parameters. According to Equation (4), each parameter modulates the input impedance by changing the equivalent inductance, capacitance, or transmission phase, thereby affecting the energy harvesting performance of the MS.

Figure 6a,b show that the inner and outer ring radii are the primary determinants of the resonant characteristics of the MS. Adjusting the radii directly alters the inductance and capacitance distribution of the unit, thereby tuning its resonant frequency. This behavior is consistent with the equivalent circuit impedance expression in Equation (7). Figure 6c,d indicate that variations in the thicknesses of the dielectric layers h_1_ and h_2_ both induce a shift in the optimal resonant frequency, with the influence of h_1_ being more pronounced. This can be attributed to the fact that the material corresponding to h_1_ (PI) possesses a higher dielectric constant. According to the expressions for the phase constant Equation (6) and characteristic impedance Equation (5) of the dielectric, variations in the h_1_ thickness exert a stronger influence on the phase constant of electromagnetic wave propagation, leading to a more significant modulation of the resonant frequency. Figure 6e,f illustrate the influence of the structural parameters of the patch at the slit. Compared to parameter m, variations in parameter n exert a more pronounced effect on the resonant frequency of the MS. This behavior is attributed to the fact that the patch region can be modeled as a parallel-plate capacitor, where n corresponds to the equivalent plate separation and thus plays a dominant role in determining the capacitance. Figure 7a,b analyze the impact of the bottom feeding structure. The length of the microstrip line and the integrated load resistance jointly determine the impedance-matching condition between the system and the load. According to the equivalent circuit model, both parameters directly influence the efficiency of energy coupling from free space to the load. The analysis of the harvesting performance under different parameters successfully validates the derived MS impedance expression.

## 3. Simulation Analysis of Metasurface

The functionality of the proposed MS was analyzed through full-wave electromagnetic simulations with a CST Microwave Studio.

### 3.1. Normal Incident Analysis

**Figure 8:**
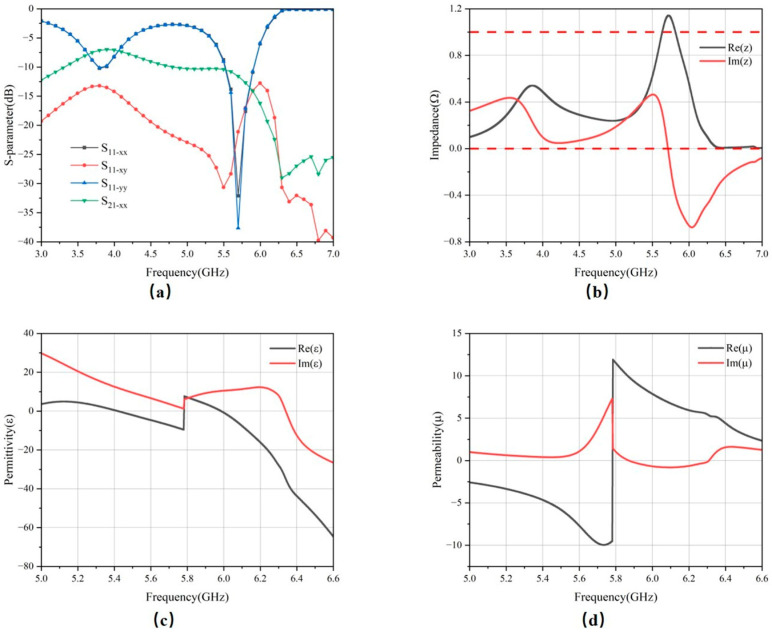
(**a**) S-parameters, (**b**) equivalent impedance, (**c**) equivalent dielectric constant, and (**d**) equivalent magnetic permeability.

The simulated S-parameters are presented in Figure 8a. In the 5.8 GHz band, the reflection coefficients S_11_ of both polarization modes are below −20 dB, indicating efficient absorption of the incident energy with minimal reflection. Additionally, the cross-polarized reflection remains below −15 dB across the band, suggesting negligible polarization conversion and thus demonstrating the structure’s polarization stability. The transmission coefficient S_21_ remains below −10 dB at the band. This limited transmission is primarily attributed to the gaps in the CPW structures on the ground plane, which result in wave leakage.

The simulated effective electromagnetic parameters—equivalent permittivity, permeability, and normalized impedance—are presented in Figure 8c,d. At 5.8 GHz, the real and imaginary parts of both the equivalent permittivity and permeability are negative, classifying the structure as a double-negative (DNG) metamaterial. This anomalous electromagnetic response is the fundamental mechanism behind its strong absorption. Furthermore, as shown in Figure 8b, the real part of the equivalent input impedance approaches unity, while its imaginary part approaches zero, indicating good impedance-matching across this frequency band.

**Figure 9:**
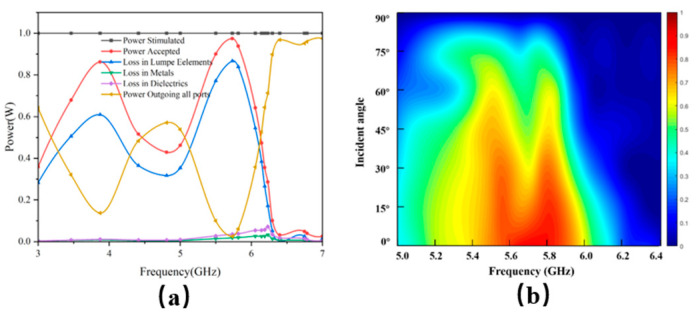
(**a**) Energy distribution and (**b**) collection efficiency with incident angle.

The efficiency of transferring the absorbed energy to a resistive load is a crucial performance metric for MS. The HE η is quantified using the following expression:

(9) $$\eta =\frac{P_{\mathrm{Load}}}{P_{in}}\times 100\%$$

where P_Load_ represents the power received by the load resistor and Pin denotes the total power incident on the MS. Figure 9a presents the electromagnetic energy distribution, showing a peak HE of 86.6% at 5.72 GHz. Furthermore, Figure 9b displays the variation in HE under different angles of incidence. The results demonstrate that the MS maintains an efficiency exceeding 50% across an incidence angle range of 0° to 60°, confirming its wide angular harvesting capability.

**Figure 10:**
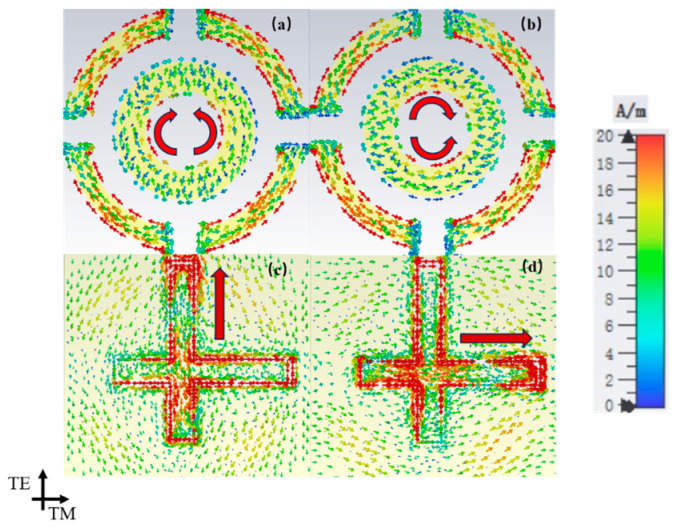
Variation in surface current density with polarization angle. (**a**) Upper φ = 0°, (**b**) upper φ = 90°, (**c**) lower φ = 0° and (**d**) lower φ = 90°.

**Figure 11:**
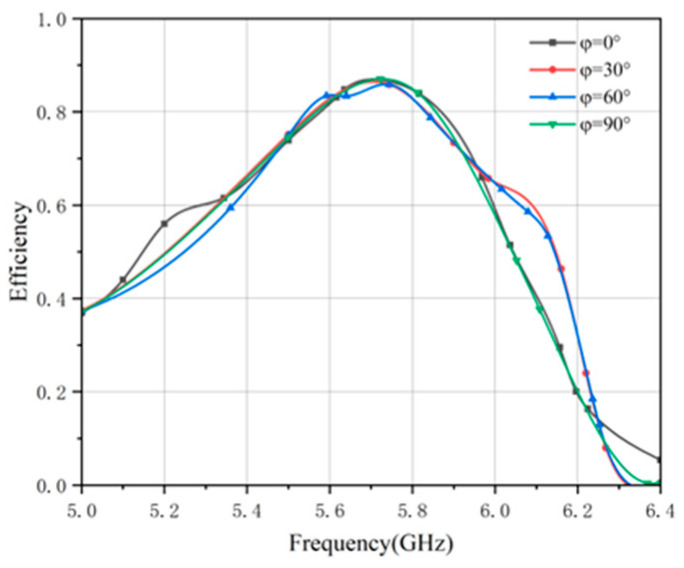
Harvesting efficiency of MS at different polarization angles.

To further investigate the energy harvesting mechanism at the absorption peak, the surface current distribution was analyzed. As shown in Figure 10, under a normal wave incidence, currents are induced along the polarization direction on the top-layer split-ring element, demonstrating effective capture of the incident wave. This energy is subsequently coupled with the CPW structure on the bottom surface, forming loop currents along the microstrip line aligned with the incident wave’s polarization. The currents then converge toward the port, consistently with the polarization mode, and are ultimately collected by the load resistor. It can be observed that the charge accumulates at the T-shaped outer ring and the inner ring parallel to the polarization direction, which is attributed to the equivalent capacitance in these regions. Figure 11 also shows the HE under different polarization angles. The minimal variation in efficiency with the polarization angle confirms the polarization-insensitive nature of the MS.

Analysis of the surface current distributions across the metal layers elucidates the efficient microwave absorption mechanism of the proposed resonant unit. Moreover, it verifies that embedding the CPW feedline into the bottom metal layer successfully couples the electromagnetic energy absorbed by the MS to the load resistor. This coupling mechanism is well-suited for flexible electromagnetic MS designs, thereby providing a foundation for their application in bendable devices.

### 3.2. Conformal Incidence Analysis

**Figure 12:**
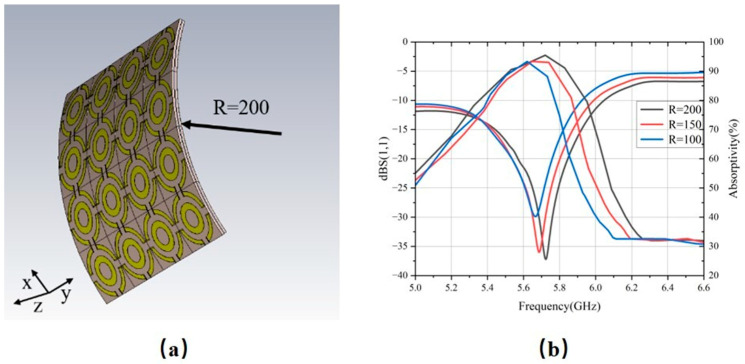
(**a**) MS in a bent configuration and (**b**) reflection coefficient and absorption efficiency of MS under different bending radii.

To evaluate the flexibility of the designed MS, a 4 × 4 unit-cell array was simulated under various bending radii to observe its reflection coefficient and absorption rate, as shown in Figure 12. As the bending curvature increases, the minimum of S_11_ shifts from −36 dB at 5.72 GHz to −27 dB at 5.66 GHz, accompanied by a drop in the absorption rate from 95.5% at 5.72 GHz to 92.3% at 5.62 GHz. This phenomenon is attributed to the deformation of the unit cells caused by bending and the change in the normal component of the incident wave vector. These factors collectively alter the impedance-matching between the MS and free space, resulting in a decline in absorption efficiency and a shift in the resonant frequency.

## 4. Experimental Validation

**Figure 13:**
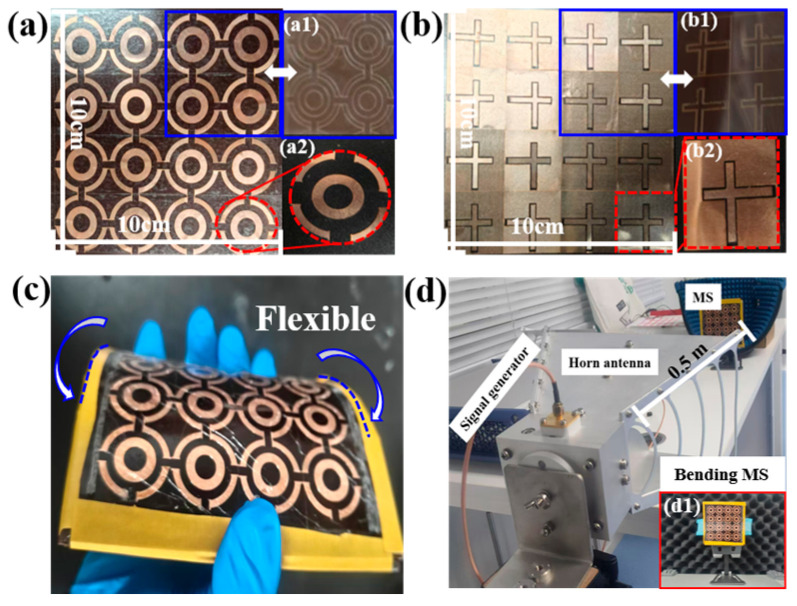
Prototype of the 4 × 4 flexible MS (**a**) top and (**b**) back views of the assembled prototype, with insets showing the fabrication steps: (**a1**,**b1**) laser marking of the copper patch pattern on the PI substrate, and (**a2**,**b2**) a single copper patch unit cut by laser. (**c**) Flexible states, and (**d**) measurement system, with (**d1**) depicting the MS in its bending test state.

To validate the performance of the designed MS, a 4 × 4 MS array was fabricated for testing, as depicted in Figure 13. The copper patches were cut using a 1070 nm, 175 W fiber laser, while their positions on the PI substrate were etched using a 1030 nm, 4.5 W fiber laser. Then, the copper patches and PI substrate are bonded together by PI double-sided tape. The overall aperture is 100 mm × 100 mm, comprising a 0.05 mm-thick copper layer bonded to a 1 mm-thick polyimide substrate. The measurement setup is shown in Figure 13. It consisted of a linearly polarized transmitting antenna placed on a rotatable holder 0.5 m away from the MS, an Agilent E8267D Signal Generator as a power source, and a Rohde Schwarz NRP-Z55 thermal power sensor, used to measure the output power at the load. By rotating the transmitting antenna, the angle of the incident wave is changed from 0° to 60°. In addition, the MS structure is conformally wrapped around cylinders of different radii to evaluate its harvesting performance under bending. The power captured by the MS array is recorded using a calibrated power meter to quantify the variation in HE with the incident angle and curvature.

**Figure 14:**
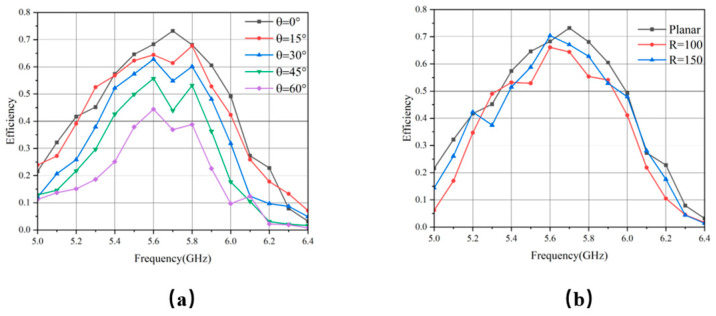
HE (**a**) under different incident angles and (**b**) under different conformal radii.

Based on experimental results, the RF-to-AC conversion efficiency of the proposed MS across the frequency and incident angle is summarized in Figure 14a. Under normal incidence, a peak HE of 73.24% is achieved at 5.7 GHz. As the incident angle increases, the efficiency decreases gradually, accompanied by a shift in the optimal frequency. This trend is attributed to the degraded impedance-matching between the MS and free space at oblique angles, resulting in a more rapid efficiency roll-off. Nevertheless, the structure retains a HE above 40%, even for angles exceeding 45°, demonstrating robust wide-angle performance.

A quantitative comparison with state-of-the-art MS harvesters is provided in Table 1. The proposed design simultaneously offers polarization insensitivity, wide-angle stability, mechanical flexibility, and competitive RF-to-AC efficiency, outperforming most reported flexible counterparts.

Additionally, we evaluated the energy HE of the MS under various conformal curvature radii R. As illustrated in Figure 14b, when R is 150 mm and 100 mm, the maximum HE remains above 65%, albeit with some frequency shift. These results indicate that the designed MS array retains excellent energy harvesting performance across various curvatures.

It should be noted that discrepancies were observed between the experimental and simulation results, which can be attributed to the following factors: (i) the simulation assumes an ideal infinite array with periodic boundary conditions, whereas the experiment employs a finite 4 × 4 MS energy harvester, leading to edge diffraction effects; (ii) material substitution, where replacing the idealized central air gap with a polymethacrylimide (PMI) foam spacer for structural support introduces dielectric loading, which shifts the resonant frequency, causing an efficiency drop at the target frequency; (iii) manufacturing tolerances and assembly misalignment, where laser-cutting deviations in critical geometric parameters, such as the T-shaped gaps, perturb the local resonance conditions, while manual layer-to-layer alignment offsets weaken the electromagnetic coupling between the top resonator and the bottom feeding layer; and (iv) parasitic losses from SMA connectors and manual soldering joints that were not accounted for in the idealized simulations. Together, these practical factors comprehensively account for the observed reduction in HE.

## 5. Conclusions

This study presented an efficient flexible MS electromagnetic energy harvester operating at 5.8 GHz. The proposed MS unit evolves from a conventional dual-ring resonator. It comprises a dual-ring structure with a T-shaped segmented outer ring and is integrated with a CPW on the back side. This configuration efficiently directs the captured electromagnetic energy to a resistive load, achieving a simulated energy HE of 86.6%. The physical mechanism was elucidated through an equivalent circuit model, which confirmed that the high efficiency stems from precise impedance-matching between the MS and free space. The surface current analysis confirmed effective energy capture across different polarization angles. Furthermore, absorption efficiency simulations under various bending radii confirmed its operational reliability in curved states. The experimental results demonstrated a maximum HE of 73.2% in the planar state, which remained above 40%, even at a 45° oblique incidence angle. When conformally attached to cylindrical surfaces with different radii of curvature, the HE was consistently maintained above 65%. In summary, the proposed design not only exhibits high efficiency, polarization insensitivity, and wide-angle incidence adaptability, but also possesses excellent flexible conformal characteristics. To further ensure stable operation in dynamic wearable scenarios, the MS can be integrated with a broadband rectifier and an energy storage buffer to effectively smooth out efficiency fluctuations caused by varying bending conditions. This robust and compact MS harvester provides a reliable power solution for low-power non-planar devices, such as biomedical electronics and wearable systems.

## Figures and Tables

**Table 1:** Comparison with other energy harvesters.

Ref.	Fre. (GHz)	Unit Size	Mat.	Flex.	HE
[27]	2.6	0.22λ × 0.22λ	F4B	No	83%
[28]	5.8	0.27λ × 0.27λ	PTFE	No	68%
[29]	5.8	0.2λ × 0.2λ	PB260	Yes	54%
[30]	5.8	1.17λ × 1.17λ	PI	Yes	78%
This work	5.72	0.48λ × 0.48λ	PI	Yes	86.6%

## Data Availability

The original contributions presented in this study are included in the article. Further inquiries can be directed to the corresponding author(s).
